# The impact of students’ mathematical attitudes on intentions, behavioral engagement, and mathematical performance in the China’s context

**DOI:** 10.3389/fpsyg.2022.1037853

**Published:** 2022-11-29

**Authors:** Limei Wang, Fuqiang Peng, Naiqing Song

**Affiliations:** ^1^School of Science, West Yunnan University, Lincang, Yunnan, China; ^2^School of Mathematics and Statistics, Southwest University, Chongqing, China; ^3^School of Foreign Languages, West Yunnan University, Lincang, Yunnan, China; ^4^Centre for Collaborative Innovation of Assessment Toward Basic Education Quality, Southwest University, Chongqing, China

**Keywords:** attitude, subjective norms, perceived behavioral control, intentions, behavioral engagement, mathematical performance

## Abstract

Referring to the theory of planned behavior (TPB), this study intends to investigate the impact of students’ mathematical attitude determinants (i.e., attitude, subjective norms, and perceived behavioral control) on intentions, behavioral engagement, and mathematical performance. The data collected online in China’s context and the research hypotheses are developed and then tested through structural equation modeling. It is found that attitude and subjective norms have effects, directly or indirectly, on intentions, behavioral engagement, and mathematical performance. In addition, the intentions have a significant effect on behavioral engagement, and behavioral engagement does likewise on mathematical performance. It has also been accepted that perceived behavioral control is not directly related to intentions but largely to behavior and indirectly to mathematical performance through behavior alone. In conclusion, this study’s findings will contribute to the current literature on mathematical performance and will also inform the policymakers of the proposal on students’ mathematics belief and attitude interventions as a means to improving students’ mathematical performance.

## Introduction

Mathematical performance refers to an individual’s capacity to reason mathematically and to solve mathematical problems through formulation, employment, and interpretation of mathematics in the diverse contexts of the real world (OECD, 2018). The performance has received, as part of the core test contents of the Program for International Student Assessment (PISA), wide attention from the educational community (Cogan et al., 2019; Gjicali and Lipnevich, 2021). Mathematics is considered the key to promoting the development of the cognitive domain of students and is fundamental to students’ future development and communication (Lavidas et al., 2022). Developing a strong foundation in early math skills is vital for children’s later educational success and economic, health, and employment outcomes (Papadakis et al., 2016a). Children who enter school with strong mathematics skills have a greater likelihood of success in mathematics in kindergarten and later grades (Papadakis et al., 2021). Furthermore, mathematical performance can be a pivotal reference for student admission to higher institutions or well-paid career opportunities, as an indicator of competitiveness to meet the demands of economic globalization (Burrus and Moore, 2016). Longitudinal research indicates that low attainment in mathematics can have significant long-term consequences, affecting later school achievement, employment, criminality, mental health, and future earnings (Papadakis et al., 2016b,^2018^). Particularly, in China, since the PISA 2012 Assessment and Analytical Framework in Shanghai came out among the top ones (OECD, 2013), math education has been drawing enormous attention and a research boom. The outcome also reproves that students from many other PISA member countries do not perform at expected levels in mathematical performance assessment (Fleischman et al., 2010). Hence, to predict students’ mathematical performance, test factors can be essential for educational policy-making and practice.

From the literature available on mathematical performance, most studies focus on three dimensions, demographic (e.g., gender, socioeconomic status perspectives, and family resources) (Lopez et al., 2007; Thien, 2016; Kang and Cogan, 2020), cognitive (e.g., classroom context, teacher expectancy effects, school-level factors, and new technologies) (Papadakis et al., 2016a, 2018; Lazarevic and Orlic, 2018; Trusz, 2018; Kitsantas et al., 2021), or non-cognitive (e.g., anxiety, self-efficacy) (Niepel et al., 2018; Hiller et al., 2022). However, from the perspective of educational psychology, some studies relate non-cognitive constructs of beliefs and attitudes to mathematical performance, which has largely remained seldom explored (Burrus and Moore, 2016). Most previous studies concentrated on non-cognitive predictors of mathematical performance, mainly discussing student self-efficacy (Skaalvik et al., 2015; Kurniawati and Mahmudi, 2019), confidence in mathematics (Stankov et al., 2012, 2014), and motivational constructs (Garon-Carrier et al., 2016; Ker, 2017; Smart and Linder, 2018). Although some of the studies examined, longitudinally or cross-sectionally, the predictive value of mathematics beliefs and attitudes by referring to the theory of planned behavior (TPB) framework (Lipnevich et al., 2011; Niepel et al., 2018), there is still limited research relying on the predictive value of other non-cognitive factors such as attitudes and beliefs on mathematical performance. Recently, based on the PISA 2012 public-use data in the United States, Gjicali and Lipnevich (2021) investigated the impacts of students’ mathematical attitudes on the intentions to study mathematics, behavioral engagement, and mathematical performance. In the extant literature, however, educational studies of the predictive roles of non-cognitive factors, such as attitudes and beliefs on mathematical performance, have not yet utilized China’s data, still lacking the vast, complex, and diverse Chinese context.

Unlike prior studies treating behavioral engagement as a proxy for mathematical performance (Burrus and Moore, 2016; Niepel et al., 2018), this study modifies and extends the TPB framework applying one more component of mathematical performance and exploring specific academic behaviors as predictors of mathematical performance. This study investigates the correlation between students’ mathematical attitudes and behavioral engagement. It also explores the mechanisms for a holistic understanding of the impact of students’ mathematical attitudes on mathematical performance using the data collected in China. Specifically, we try to update all the mathematical attitude determinants (attitude, subjective norms, and perceived behavioral control) under the term “TPB-based students’ mathematical attitudes.”

This paper contributes four aspects. First, empirically it explores the effect of students’ mathematical attitudes on mathematical performance by referring to the data collected in China. It also extends the studies on students’ mathematical attitudes, in addition to previous ones mainly with the OCED member countries. Second, this paper, in the updated TPB structure, applies the modified model by introducing the mathematical performance, which is differentiated from behavioral engagement. Presuming behaviors as independent from mathematical performance, this study achieves a more accurate understanding of the behavior-literacy relevance in educational studies. Third, it examines, directly and indirectly, the effects of the attitude-specific determinants that are likely to influence intentions, behavioral engagement, and mathematical performance. Then, it goes on the probe into whether the intentions affect mathematics behavioral engagement and mathematical performance while trying to discover the correlation between mathematics behavioral engagement and mathematical performance. Fourth, the findings of this study may provide further support for their application in the domain of mathematics education. Also, for the sake of the policymakers and mathematics education researchers’ reference to devise future mathematical attitude interventions, such extensive studies from the perspective of educational psychology would be of any implications.

The paper is structured as follows. Section “Theoretical background and hypothesis development” commences with the theoretical background and reviews the literature to conceive the research hypotheses and test them in this study. Section “Methodology” furnishes the sample and methodology to test these hypotheses. Section “Results” releases the empirical results. Section “Discussion” discusses the results and boils them down to findings. Finally, based on the findings, the conclusions are drawn in Section “Conclusion.”

## Theoretical background and hypothesis development

### The theory of planned behavior

The TPB framework, which was coined by an American researcher Ajzen (1991), has its root in the theory of reasoned action (TRA). In light of TRA, subjective norms and behavioral attitudes are deemed as the driving factors that affect behavioral intention. With perceived behavioral control introduced as a factor, TRA was developed into a new, planned behavior theory research model, namely TPB. The model takes behavior intention as the directest influencing factor for behavioral engagement, while such intention is subject to attitude, subjective norms, and perceived behavioral control.

In the disciplines of psychology, management, and sociology, as well as the arenas of political participation and environmental protection, among others, researchers have used the TPB model to predict and explore the causal factors of different human behaviors in several approaches (Cooper et al., 2016), such as technological application (Rai et al., 2002), voluntary participation (Dawkins and Frass, 2005), examination of intention and entrepreneurial behavior (Kautonen et al., 2013), environmental conservation (Wauters et al., 2010), exercise of behaviors (Ickes and Sharma, 2012), and sleep patterning (Knowlden et al., 2012). At the same time, the TPB model has also been deployed to a possibly holistic extent within the field of studies on education. The model highlights the linkage of the instructional intentions of the prospective science teachers with their awareness and experience of science in their educational studies (Cooper et al., 2012). It is also used to predict teacher behavior, such as teacher development (Dunn et al., 2018), teacher entrepreneurship (Yang and Zhao, 2019), and technology-enabled learning (Watson and Rockinson-Szapkiw, 2021), all being intention-specific. In addition, the TPB model has been proposed to facilitate the understanding of student behavior and achievement (e.g., Cooper et al., 2016), such as student entrepreneurial intention (Wang et al., 2021), online interactive behaviors (Pan et al., 2021), mathematical performance (Lee and Stankov, 2018; Gjicali and Lipnevich, 2021), and mobile learning (Azizi and Khatony, 2019).

Through this study, we extend the TPB framework to explore the effects of students’ mathematical attitudes on mathematical performance and deem specific behavioral engagement as a predictor of mathematical performance. In the TPB framework, the three students’ mathematical attitude determinants are designated as attitude, subjective norms, and perceived behavioral control, to hypothesize and predict behavioral intention, behavioral engagement, and mathematical performance. The intention is deemed as a mediator among the students’ mathematical attitude determinants and behavior. Furthermore, the TPB framework postulates that perceived behavioral control also has an indirect or mediated effect on behavioral engagement, through intention.

### The theory of planned behavior-based students’ mathematical attitudes and intention to pursue mathematics

The TPB-based students’ mathematical attitudes comprise constructs, attitude, subjective norms, and perceived behavioral control, for the prediction of the intention (Ajzen, 1991). Attitude can be defined, by the TPB model, as the perceived evaluation of the consequences and behavioral characteristics, positive or negative (Azizi and Khatony, 2019). The subject norm implies an individual belief in the importance of people’s thinking about the specific behavior and their act (Ajzen, 2005). Perceived behavior control indicates an individual perception level to perform a behavioral effort while measuring the individual control over behavior (Yeap et al., 2016).

Prior studies also suggest that the behavior of individuals may be strikingly affected by their confidence (Bandura et al., 1980). Through their experimental studies of student classroom attendance, Ajzen and Madden (1986) found that, after factoring in the mathematical attitude, determinants may act as a significant predictor of intention. A meta-analysis of the TPB efficacy also indicates that the attitudes, norms, and control may account for 39% of behavioral intention variants (Armitage and Conner, 2001). Recently, by sampling various US high schools, Niepel et al. (2018) identify the positive association of students’ intention to succeed in mathematics with the aforesaid mathematical attitude determinants.

Therefore, we intend to test the following hypotheses:

H1: The significant effect of students’ attitudes on their intentions to pursue mathematics.

H2: The remarkable effect of subjective norms on the same intentions.

H3: The noticeable effect of students’ perceived behavioral control on identical intention.

### Students’ mathematical attitudes, intentions, behavioral engagement, and mathematics performance

An intention implies to be one’s willingness to exert a certain behavior (Gjicali and Lipnevich, 2021). Ajzen (2005), in his studies, proved a significant attribution of the factor of volitional behavior to an individual’s intention of engagement in that behavior. Furthermore, based on their survey of some 15-year-old students Hagger et al. (2015), documented that, while intentions of mathematics learning predict that for the grades and homework behavior in the discipline, perceived behavioral control directly did the same to the outcomes of both factors. There is evidence suggesting that TPB-based mathematical beliefs and attitudes may herald mathematical performance and achievement (Simzar et al., 2015). Other prior studies have also demonstrated the conceptional relevance of perceived behavioral control to self-efficacy beliefs (Bandura, 1977). Furthermore, based on the survey of the Grade X junior high school students, Simzar et al. (2015) see that mathematics self-efficacy predicts mathematics achievement.

Some studies indicate that a great deal of academic behaviors constitutes some behavioral factors contributing to academic achievements (Gjicali and Lipnevich, 2021). By sampling 34 countries that ever participated in the PISA 2012, Fung et al. (2018) concluded that student participants in more varied mathematics behavior promise higher levels of mathematics achievement. However, a survey of 14,000-plus student participants in the OECD’s PISA, Australia, revealed that the TPB-based students’ mathematical attitudes turn out to be poor predictors of mathematical intentions and mathematical behavior (Skrzypiec and Lai, 2017). Moreover, the PISA 2012 evidence demonstrates that students’ attitudinal beliefs (e.g., dispositional, normative, and control beliefs) about mathematics (Areepattamannil et al., 2016), perceived behavioral control toward mathematics (Karakolidis et al., 2016), and high subjective norms (Areepattamannil et al., 2015) are, unexceptionally, all associated with mathematics behaviors and mathematical performance.

One recent study differentiated students’ behaviors as an independent factor from mathematical performance, treating behavioral engagement as a predictor of mathematics achievement (Gjicali and Lipnevich, 2021). In the PISA, mathematical performance is gauged with test questions for mathematics literacy, access to mathematical reasoning and tools in personal and professional contexts (OECD, 2018).

Therefore, we intend to test the following hypotheses:

H4: The direct effect of students’ perceived behavioral control over mathematics behavioral engagement.

H5: The significant effect of their intention to pursue mathematics on mathematics behavioral engagement.

H6: The significant effect of their mathematics behavioral engagement on mathematics performance.

H7: The indirect effect of their perceived behavioral control over mathematics on mathematical performance through behavioral engagement.

H8: The indirect effect of their mathematical attitudes, subjective norms, and perceived behavioral control, respectively, on mathematical performance through intention and behavioral engagement.

**FIGURE 1:**
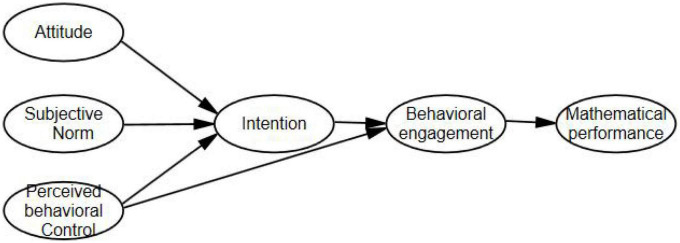
Conceptual framework.

In conclusion, to string these relationships up, we have developed a conceptual model (refer to Figure 1).

## Methodology

### Sample and data collection

In this study, we select Grade 9 junior high school students as the study subjects. Data were collected using online tests and online questionnaires. All participants voluntarily agreed to take part in the study. As they were all minors, their guardians approved their participation in the study by signing the research information letter. All students who participated in the test entered the computer classroom and utilized the test platform under the guidance of the teacher. Before the actual research, a pre-study was carried out with some Grade 9 high schoolers in Yunnan Province of China, with the questions revised repeatedly into the formal questionnaire. The survey proceeded from 5 January 2022 to 28 February 2022, the sample being used a random sampling method and mainly taken from Yunnan Province, Guizhou Province, Guangxi Province, Guangdong Province, and Shandong Province of China. A total of 405 questionnaires were returned, and 326 were deemed valid with a return rate of 80.49%, exclusive of the incomplete and invalid ones. The assessed subjects average 15.81 years of age, 53.4% of whom are boys and 46.6% girls, at a male-to-female ratio of 1:0.87. After the sampling and data collection, the research data are processed and analyzed using SPSS26.0 and AMOS24.0.

### Variables and measurement

To ensure the reliability and validity of the measurement instrument, this study intends to apply, as far as possible, well-established scales in the existing literature, with appropriate modifications for the purpose of the measurement questions. Ratings of attitude, subjective norms, perceived behavioral control, and behavioral engagement are based on a 4-point Likert scale, by which the variables are scored 1 up to 4 points (1 = strongly agree, 2 = agree, 3 = disagree, and 4 = strongly disagree). Specifically, the five items of the intention factor are measured, by forced choice, adapted from OECD (2014) and Gjicali and Lipnevich (2021); six items of the subjective norms factor are scaled, according to OECD (2014); six items of the perceived behavioral control factor are included, with reference to Gjicali and Lipnevich (2021); and eight items of the behavioral engagement are examined in Fung et al. (2018). In addition, the high school student subjects’ average scores on the mid- and final-semester exams are referred to as mathematical performance.

## Results

### Tests for reliability and validity

For the sake of assessment of the reliability of the variables on the measurements, we adopt Cronbach’s alpha to test for their internal consistency, and by confirmatory factor analysis, we intend to demonstrate convergent validity by adopting SPSS 26.0. As shown in Table 1, Cronbach’s alpha coefficient for each measuring dimension is greater than 0.8, suggesting good reliability for the aforesaid sampling data.

**TABLE 1:** Reliability and convergent validity.

Constructs	Test items	Factor loading	Cronbach’ α	CR	AVE
Attitude	Trying hard at school will help me secure a good job.	0.759	0.827	0.833	0.555
	Trying hard at school will help me be admitted to a good college.	0.807			
	I enjoy being rated high grades.	0.685			
	Trying hard at school matters.	0.724			
Subjective Norms	Most of my friends do well in math.	0.746	0.867	0.859	0.505
	Most of my friends work hard at math.	0.746			
	My friends encourage me to take math tests.	0.648			
	My parents believe that it’s important for me to study math.	0.682			
	My parents believe that math is important for my career.	0.718			
	My parents like math.	0.717			
Perceived behavioral control	I can succeed in math learning with enough efforts.	0.820	0.907	0.910	0.629
	Doing well in math is completely up to me.	0.771			
	If I wanted to, I can perform well in math learning.	0.805			
	My family demands me to work out math problems.	0.791			
	I have different math teachers.	0.761			
	I perform poorly in math learning regardless of efforts.	0.808			
Intention	I intend to take additional math courses after school.	0.790	0.842	0.872	0.576
	I plan to take a major in college that requires math skills.	0.715			
	I am willing to study harder in math classes than the course requires.	0.731			
	I plan on taking as many math classes as I can during my schooling.	0.757			
	I plan to pursue a career that involves much math learning.	0.799			
Behavioral engagement	I finish my assignment in time for math class,	0.829	0.941	0.936	0.646
	I work hard at my math homework.	0.814			
	I am preparing for my math exams.	0.753			
	I keep studying until I understand math material.	0.814			
	I have the motivation to attend the math class.	0.820			
	I listen to the teacher attentively in math class.	0.734			
	I avoid distractions when I am studying math.	0.788			
	I keep my math work well-structured.	0.871			

To assess the validity of the variables in the measurements, this study explored convergent validity and discriminative validity. In particular, convergent validity is determined by composite reliability (CR) and average variance extracted (AVE). Also, if the square root of the AVE of a variable is greater than the correlation coefficient of that variable with the other ones, the differential validity can be assessed as good. As shown in Table 1, the CR and AVE values for this study are greater than the standard values of 0.8 and 0.5, respectively, indicating good convergent validity of the scale (Nunnally, 1978). In addition, coefficients greater than 0.6 for each dimensional measure may effectively reflect the potential traits of their corresponding dimensions. In Table 2, the AVE open root value for each latent variable is greater than the correlation coefficient between that latent variable and the other latent variables. Therefore, the measurement model is of differential validity (Fornell and Larcker, 1981). To sum up, the findings of this study show that the efficiency and integrity of each build are satisfactory.

**TABLE 2:** Validity of potential variables.

Constructs	Mean	Std. D	Attitude	Subjective norms	Perceived behavioral control	Intention	Behavioral engagement
Attitude	3.273	0.966	**0.745**				
Subjective norms	3.456	0.822	0.466**	**0.711**			
Perceived behavioral control	3.648	0.847	0.324**	0.432**	**0.793**		
Intention	3.255	0.850	0.315**	0.370**	0.263**	**0.759**	
Behavioral engagement	3.329	0.999	0.475**	0.475**	0.336**	0.252**	**0.804**

*The diagonal bold text stands for the open root value of the AVE. **P < 0.01.

### Model fit test

Model fit is the degree of consistency between the theoretical model and the sample model. AMOS 24.0 is used to test the model’s goodness of fit. The goodness-of-fit index (GFI), adjusted goodness-of-fit index (AGFI), relative fit index (TLI), and comparative fit index (CFI) are all greater than 0.9; the closer to 1.0 suggests the better goodness of fit between the data and the model, and the greater than 0.8, an acceptable model. Provided that the variability index RMSEA is less than 0.080, the model is assessed as a good fit (Wu, 2000).

The test results are shown in Table 3, χ^2^/df = 1.484, GFI = 0.903, AGFI = 0.885, TLI = 0.962, CFI = 0.965, and RMSEA = 0.039. Therefore, the sample model has good goodness of fit.

**TABLE 3:** Fit indices of measurement and structural model.

Fit index	χ^2^/d*f*	GFI	AGFI	TLI	CFI	RMSEA
Reference value	<3	>0.9	>0.8	>0.9	>0.9	<0.08
Examined value	1.484	0.903	0.885	0.901	0.962	0.039

### Hypothesis testing

The results of the path relationship are illustrated in Table 4. As predicted, the attitudes are related positively to student intentions to pursue mathematics (β = 0.203, *SE* = 0.071, *p* = 0.01), and so we interpret the result as supportive of H1. The subjective norms significantly influence their intentions to pursue mathematics (β = 0.293, *SE* = 0.117, *p* < 0.01), and thus, the result is considered supportive of H2. Similarly, the perceived behavioral control is related positively to behavioral engagement (β = 0.309, *SE* = 0.075, *p* < 0.01), and therefore, the result is deemed supportive of H4. However, the perceived behavioral control does not significantly affect their intention to pursue mathematics (β = 0.084, *SE* = 0.070, *p* = 0.226), and consequently, the result is found to be non-supportive of H3. In addition, the intention to pursue mathematics significantly affects mathematics behavioral engagement (β = 0.211, *SE* = 0.076, *p* < 0.01), and accordingly, the result is seen as supportive of H5. The mathematics behavioral engagement is related positively to mathematical performance (β = 0.348, *SE* = 0.747, *p* < 0.01), which hence leads to the acceptance of H6.

**TABLE 4:** Test results of path relationship.

Hypothesized relationship	*B*	*SE*	*t-*value	*P-*value	Supported?
Attitude– > Intention	0.203	0.071	2.593	0.01	Yes
Subjective norms– > Intention	0.293	0.117	3.431	<0.001	Yes
Perceived behavioral control– > Intention	0.084	0.07	1.211	0.226	No
Intention– > Behavioral engagement	0.211	0.076	3.431	<0.001	Yes
Perceived behavioral control– > Behavioral engagement	0.309	0.075	5.124	<0.001	Yes
Behavioral engagement– > Mathematical performance	0.348	0.747	6.422	<0.001	Yes

The results of the mediation analysis are specified in Table 5. We find that perceived behavioral control has an indirect effect on mathematical performance through behavioral engagement (*p* = 0 < 0.01, 95% CI [0.065, 0.167]), and thus, the result indicates that H7 applies. In addition, the attitudes have an indirect effect on mathematical performance through intention and behavioral engagement (*p* < 0.05, 95% CI [0.003, 0.041]). Similarly, subjective norms have an indirect effect on mathematical performance through intention and behavioral engagement (*p* < 0.05, 95% CI [0.006, 0.051]). However, perceived behavioral control has indirect effects on mathematical performance through intention and behavioral engagement (*p* = 0.178, 95%CI [–0.004, 0.023]). Therefore, the result can be inferred as partially supportive of H8.

**TABLE 5:** Test results of mediation analysis.

Hypothesized relationship	Estimate	95% CI	*P-*value	Supported?
Perceived behavioral control– > Behavioral engagement– > mathematical performance	0.108	[0.065, 0.167]	0	Yes
Attitude– > Intention– > Behavioral engagement– > Mathematical performance	0.015	[0.003, 0.041]	0.011	Yes
Subjective norms– > Intention– > Behavioral engagement– > Mathematical performance	0.022	[0.006, 0.051]	0.04	Yes
Perceived behavioral control– > Intention– > Behavioral Engagement– > Mathematical performance	0.006	[–0.004, 0.023]	0.178	No

**FIGURE 2:**
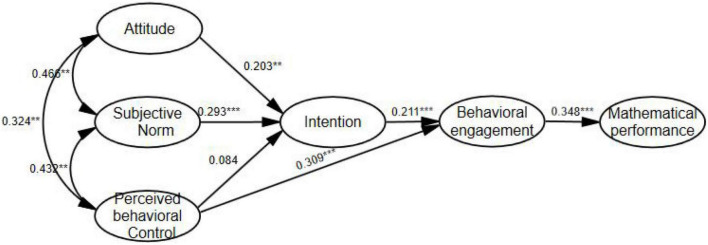
Path relationships.

After the above path analysis and hypothesis testing, the specific path relationships between students’ mathematical attitudes, intentions, behavioral engagement, and mathematical performance in the research model are shown in Figure 2.

## Discussion

The results of our study show that students’ attitude and subjective norms affect their intentions to pursue mathematics positively to a great extent (H1 and H2). Similar to the results Lipnevich et al. (2016), hypothesize that the students’ attitudes toward mathematics have the strongest correlation to intentions. Hagger et al. (2015) also find that attitude and subjective norms predict intention. Contrary to one of the aforesaid hypotheses, we do not find perceived behavioral control’s significant connection to their intentions (H3). As for the perceived behavioral control, to some extent, similar to the findings of Gjicali and Lipnevich (2021), is found indirectly related to intentions. The students’ sense of competency over accomplishing a particular action or achieving the goal does not directly affect the students’ intention. However, there are conflicting results, from considerable studies. For example, Niepel et al. (2018) argue that the student’s intention to pursue mathematics is determined by attitudes toward the behavior, subjective norms, and perceived behavioral control, which can be interrelated and might account for the different results of longitudinal designs in data collection.

Perceived behavioral control is found to affect behavioral engagement (H4). Similar studies have been conducted by Areepattamannil et al. (2016), who adopt the Qatari sample of the PISA 2012, and they also find that students’ perceived behavioral control about mathematics is associated with mathematics behaviors. In addition, behavioral engagement as a mediator between perceived behavioral control and mathematical performance is proven by this study (H7). This is consistent with the study of You et al. (2011), who together demonstrate that perceived behavioral control has an indirect effect on mathematical performance through behavioral engagement. It is noteworthy that perceived behavioral control is not directly related to intentions, but largely, to behavior and indirectly related to mathematical performance through behavior alone. The fact is that, regarding the indirect effect of perceived behavioral control on behavior, intentions are not a particularly useful factor in mediating that relation (Gjicali and Lipnevich, 2021).

The results in this study show that students’ intentions to pursue mathematics affect behavioral engagement (H5), and mathematics behavioral engagement significantly affects mathematical performance (H6). The student’s intentions to pursue mathematics-relevant coursework or careers after high school are essential for predicting their behavioral engagement (e.g., day-to-day work ethic on mathematics homework, exam preparations, and attentiveness in class) (Gjicali and Lipnevich, 2021). Fung et al. (2018) also found a positive relationship between behavioral engagement and mathematical performance. In accordance with the literature related, Robinson and Mueller (2014) found that the components of students’ engagement, affective, behavioral, or cognitive, are individually related to their mathematics achievement (2014). Additionally, some studies (Burrus and Moore, 2016; Niepel et al., 2018) apply the TPB model to interpret the variability in mathematics achievement in conflating academic behaviors and achievements.

Analyses of the mediating effects of intentions indicate that the students’ mathematical attitudes and subjective norms are found to have an indirect impact on mathematical performance through intention and behavioral engagement, whose outcome partially supports H8. Fostering students’ attitudes toward mathematics are believed to have a positive effect on academic intentions, behaviors, and achievements. The finding that subjective norms are related to the academic outcomes of interest in this study (intentions, behavior, and subsequent mathematical performance) is also found consistent with Burrus and Moore (2016) and Niepel et al. (2018). Conversely, Gjicali and Lipnevich (2021) observe that subjective norms have negative effects on outcomes, direct or indirect. Also, while we adapt subjective norms from the PISA index, Gjicali and Lipnevich practice social norms from the broader psychological literature on attitudes toward sociomathematical norms. One more consideration could be the economic and cultural factors (China vs. United States) with different results.

## Conclusion

The main purpose of this study is to focus on analyses of both the direct and indirect effects of the TPB-based students’ mathematical attitude determinants on intentions, behavioral engagement, and mathematical performance. In this study, we use an extended TPB framework with a sample of 326 junior high school students in a Chinese context, and the research hypotheses are developed and tested through structural equation modeling. The conclusions can be summarized as follows: First, the attitude determinants are of direct and indirect effects on intentions, behavioral engagement, and mathematical performance. Second, perceived behavioral control is not directly related to intentions but rather, largely related to behavior and indirectly related to mathematical performance through behavior alone. Third, the students’ intentions to pursue mathematics are found to affect behavioral engagement, and the mathematics behavioral engagement significantly affects mathematical performance.

The theoretical contribution of the study includes the following: First, the TPB is a viable theoretical framework for predicting high school students’ mathematical performance in China, and the theory is applicable to relevant educational research. Second, it extends prior literature by quantifying the relationship between mathematical attitude determinants and mathematical performance in the context of China. Third, the current study takes specific academic behaviors as predictors of mathematical performance. The literature review, as a whole, shows that previous studies have predicted educational outcomes and have treated behavioral engagement as a proxy for mathematical performance. Finally, this study can be a reference for future research to further explore mathematical attitudes and their impact on mathematical performance in other parts of the world.

The findings of the current study provide several supports for their application in the fields of educational and psychological research. Policymakers and mathematics education researchers should focus on how to develop students’ confidence in the mathematical attitude determinants to achieve a higher level of mathematical performance, such as mathematics value promotion, social pressure reduction, self-efficacy increase, and explicit instruction of effective mathematics-related behaviors. In addition, the teachers need to be trained in specific instructional strategies to enhance students’ positive attitudes and self-efficacy beliefs in mathematics. They are also expected to draw students’ attention to their growth, encourage their students to try harder, and praise the students on any progress in specific mathematical skills. Finally, policymakers can rely on extensive research in the fields of social and educational psychology to design future mathematics beliefs and attitudes interventions.

Nonetheless, this study still has some limitations. First, owing to time constraints and the sampler’s unavailability, the sample size of this study was only confined to some provinces, which could have been extended across the country, with a much larger size of samples and rigorously verified error-free data. Second, sample data from different countries or regions should be included and compared with China’s data, and it should be extended to students in the other grades of high school as well. Third, the subjectivity of measurement indicators is hardly avoidable. Although its design may reduce bias and errors to a certain extent, this study adopted domestic and international scales and conducted a pilot study before the formal investigation to minimize the impact of subjective errors. However, such errors may still inevitably exist, which will be further minimized in future studies through in-depth interviews based on the grounded theory and qualitative survey. In addition, there are quite a few influencing factors (e.g., home education, teacher level, social, cultural, and economic disadvantage) that might affect mathematical performance. This study just investigated some factors, still losing sight of many others, which can be further studied in later studies by introducing possibly sufficient variables from more diverse perspectives.

## Data availability statement

The raw data supporting the conclusions of this article will be made available by the authors, without undue reservation.

## Ethics statement

This study involving human participants were reviewed and approved by the Scientific Ethics Committee of the Academic Committee of the School of Foreign Languages, West Yunnan University. All parents/guardians signed a statement of consent authorizing the participation of their children.

## Author contributions

LW, FP, and NS conceived the idea of the study and designed the study. LW and FP were conducted the data analyses, interpreted the results, and drafted the manuscript. NS provided feedback and co-wrote the final submission. All authors contributed to the manuscript revision.
